# Comparative effectiveness of the different components of care provided in heart failure clinics—protocol for a systematic review and network meta-analysis

**DOI:** 10.1186/s13643-019-0953-4

**Published:** 2019-02-02

**Authors:** Morgan Slater, Joanna Bielecki, Ana Carolina Alba, Lusine Abrahamyan, George Tomlinson, Susanna Mak, Jane MacIver, Shelley Zieroth, Douglas Lee, William Wong, Murray Krahn, Heather Ross, Valeria E. Rac

**Affiliations:** 1 Institute of Health Policy, Management and Evaluation (IHPME), Dalla Lana School of Public Health, University of Toronto, Toronto, ON Canada; 2 Toronto Health Economics and Technology Assessment (THETA) Collaborative, Toronto General Hospital Research Institute, University Health Network, Toronto, ON Canada; 3 Heart Failure/Transplant Program, Toronto General Hospital, University Health Network, Toronto, ON Canada; 4 Department of Medicine, University of Toronto, Toronto, ON Canada; 5 Department of Medicine, University Health Network, Toronto, ON Canada; 6 Division of Cardiology, Mount Sinai Hospital, Toronto, ON Canada; 7 Toronto General Hospital Research Institute, University Health Network, Toronto, ON Canada; 8 Faculty of Medicine, The University of Manitoba, Winnipeg, MB Canada; 9 Institute for Clinical Evaluative Sciences, Toronto, ON Canada; 10 School of Pharmacy, University of Waterloo, Waterloo, ON Canada; 11 Leslie Dan Faculty of Pharmacy, University of Toronto, Toronto, ON Canada; 12 Ted Rogers Centre of Excellence in Heart Function, Peter Munk Cardiac Centre, University Health Network, Toronto, ON Canada

**Keywords:** Heart failure, Systematic review, Network meta-analysis, Comparative effectiveness

## Abstract

**Background:**

Heart failure (HF) is a complex chronic condition, leading to frequent hospitalization, decreased quality of life, and increased mortality. Current guidelines recommend that multidisciplinary care be provided in specialized HF clinics. A number of studies have demonstrated the effectiveness of these clinics; however, there is a wide range in the services provided across different clinics. This network meta-analysis will aim to identify the aspects of HF clinic care that are associated with the best outcomes: a reduction in mortality, hospitalization, and visits to emergency department (ED) and improvements to quality of life.

**Methods:**

Relevant electronic databases will be systematically searched to identify eligible studies. Controlled trials and observational cohort studies of adult (≥ 18 years of age) patients will be eligible for inclusion if they evaluate at least one component of guideline-based HF clinic care and report all-cause or HF-related mortality, hospitalizations, or ED visits or health-related quality of life assessed after a minimum follow-up of 30 days. Both controlled trials and observational studies will be included to allow us to compare the efficacy of the interventions in an ideal context versus their effectiveness in the real world. Two reviewers will independently perform both title and abstract full-text screenings and data abstraction. Study quality will be assessed through a modified Cochrane risk of bias tool for randomized controlled trials (RCTs) or the ROBINS-I tool for observational studies. The strength of evidence will be assessed using a modified Grades of Recommendation, Assessment, Development, and Evaluation (GRADE) system. Network meta-analysis methods will be applied to synthesize the evidence across included studies. To contrast findings between study designs, data from RCTs will be analyzed separately from non-randomized controlled trials and cohort studies. We will estimate both the probability that a particular component of care is the most effective and treatment effects for specified combinations of care.

**Discussion:**

To our knowledge, this will be the first study to evaluate the comparative effectiveness of the different components of care offered in HF clinics. The findings from this systematic review will provide valuable insight about which components of HF clinic care are associated with improved outcomes, potentially informing clinical guidelines as well as the design of future care interventions in dedicated HF clinics.

**Systematic review registration:**

PROSPERO CRD42017058003

**Electronic supplementary material:**

The online version of this article (10.1186/s13643-019-0953-4) contains supplementary material, which is available to authorized users.

## Introduction

Heart failure (HF) is a complex chronic condition associated with a high rate of comorbidity, significant impairment of quality of life, frequent hospitalization, progressive disability, and reduced survival [1–3]. Recent estimates suggest that HF affects one million Canadians, with at least 50,000 new patients diagnosed each year [1, 2]. HF patients have a shorter life expectancy than most cancer patients, with a median survival of 1.7 years for men and 3.2 years for women [4].

HF is also associated with an enormous economic burden. It is one of the most costly chronic conditions in developed countries [5], costing Canada an estimated $3 billion each year [6]. The majority of these costs are attributable to hospitalization and other healthcare services, medications, and missed days of work [6]. HF-related hospitalizations are one of the major drivers of avoidable admissions [5, 7–9]. Roughly 25% of patients who are hospitalized for HF are readmitted within 30 days of discharge [10]. The costs of hospital admissions related to HF alone are currently estimated to be $482 million per year; this is projected to increase to $722 million by 2030 [11].

Current Canadian guidelines recommend that HF care be provided in multidisciplinary, specialized HF clinics [12]. A number of studies have demonstrated the effectiveness of these clinics [13–18]; a systematic review and meta-analysis by Holland et al. reported significant reductions in all-cause hospital admissions, HF-related admissions, and all-cause mortality [17]. A more recent study demonstrated a significant reduction in unplanned admissions among patients treated in HF specialist clinics [18]. Although studies have demonstrated the effectiveness of HF clinic care, the optimal model for management of patients in these HF clinics is unknown [8, 19]. HF clinics are very heterogeneous in structure, population, and patient management [8, 20, 21]; this heterogeneity may considerably undermine their effectiveness [19]. Recent studies have reported variability in the effectiveness of different models of care offered in HF clinics, emphasizing the gap in knowledge and practice regarding the optimal components of HF care [22–24].

The objective of this systematic review and network meta-analysis is to determine which components of care provided in HF clinics are the most effective in reducing mortality, emergency department visits, and hospitalizations and improving patient quality of life.

## Methods

This systematic review will be designed based on the Cochrane review methods [25] and will follow the Preferred Reporting Items for Systematic Reviews and Meta-Analyses (PRISMA) statement [26]. The protocol follows the Preferred Reporting Items for Systematic Review and Meta-analysis Protocols (PRISMA-P) and checklist (Additional file 1) [27, 28] and the PRISMA extension for network meta-analyses (PRISMA-NMA) [29]. This protocol is registered with PROSPERO as CRD42017058003.

### Eligibility criteria

#### Participants

The population will consist of adult patients (18 years of age and older) who are receiving treatment for a diagnosis of HF. Studies with participants who were treated by a HF clinic prior to study enrollment will be excluded. Studies that included both eligible and ineligible participants will be excluded unless they report data for the subgroup of patients who are eligible for this study.

#### Interventions

Studies must evaluate at least one component of multidisciplinary HF clinic care (or any combination of care components) with a defined intervention [18, 30–32]. Based on Canadian and US guidelines [31, 32], we anticipate including the following interventional components: education and counseling, medication management and optimization, social/peer support, clinical monitoring, referral for diagnostic testing, referral for devices (e.g., implantable cardioverter defibrillator [ICD], cardiac resynchronization therapy [CRT]), and referral for cardiac rehabilitation. This is not an exhaustive list of care components, so we will include others that have been studied in the literature.

#### Comparator

Studies must include at least one comparator arm. Comparisons can be made between other care components or to standard care. Non-comparative studies reporting a single intervention or component of care will be excluded.

#### Outcomes

Studies will be included if they report on at least one of the following outcomes, assessed after a follow-up of 30 days or more: all-cause or HF-related mortality, all-cause or HF-related hospitalizations or emergency department visits, or health-related quality of life (HRQoL) using a validated measure or a composite end-point of any of these.

#### Study designs

This systematic review will include both randomized and non-randomized controlled trials and observational cohort studies evaluating at least one component, or any combination, of multidisciplinary HF clinic care with a defined intervention [18, 30–32]. Including both controlled trials and observational studies will allow us to compare findings between these study designs, comparing the efficacy of the interventions in an ideal context versus the effectiveness in the real world.

Study designs other than controlled trials or observational cohort studies will be excluded, as will other types of publications, including letters, commentaries, and editorials. Previous systematic reviews will be used to identify relevant studies. Studies that use duplicate study populations will be reviewed to ensure that they provide different information; otherwise, only the most recent study will be included.

#### Setting

Restrictions on the type of setting will not be imposed.

#### Language

Both English and non-English language articles will be considered. Non-English articles will be translated into English for the purpose of inclusion in the systematic review.

### Information sources

Eligible studies will be identified through a systematic, comprehensive search of the following databases: MEDLINE and MEDLINE In-Process (Ovid), EMBASE (Ovid), CINHAL (Ebsco), Cochrane Database of Systematic Reviews (CDSR), DARE (Database of Abstracts of Reviews of Effectiveness), LILACS, International Clinical Trials Registry Platform (ICTRP), Science Citation Index Expanded (SCI-EXPANDED; Web of Science), PsycINFO, and Conference Proceedings Citation Index-Science (CPCI-S; Web of Science), as well as ProQuest Dissertations and Theses Global. HF disease management programs were launched in the 1990s; as such, a database search from 1990 onward is considered sufficient. The PROSPERO registry will also be searched for all active or completed systematic reviews. Electronic database searches will be supplemented with inspection of reference lists of relevant articles and hand searching of pertinent journals.

### Search strategy

The search strategy will be designed and conducted by an information specialist experienced in systematic reviews, following the Cochrane systematic review methodology [25]. It will include MeSH and natural language terms in the following concept areas: heart failure and eligible interventions. No language restrictions will be applied. The search will be updated closer to the end of our review to ensure that the most recent eligible articles are captured. A detailed preliminary search strategy for MEDLINE (Ovid) is provided in Additional file 2. The final Medline strategy will be translated into syntax appropriate for each database used in the search.

### Data management

The results of the literature search will be collected in an EndNote library, where duplicate studies will be flagged and removed. Each reviewer will receive a copy of the library for the stepwise review process. A coding scheme will be developed and used within the EndNote software to indicate eligibility status at each stage of the screening process (i.e., title/abstract, full-text screening).

### Study selection and screening process

A stepwise review process will be utilized [26]. Using a study eligibility form (Additional file 3) based on the Data Collection Form for Cochrane Reviews [33], two reviewers will independently screen in duplicate the titles and abstracts of studies identified by the search strategy. All studies deemed eligible by either reviewer will be selected for full-text review. The same reviewers will then screen the full article text and apply the eligibility criteria stated above. During the full-text screening phase, both reviewers will record the reason for exclusion in a standardized data abstraction document based on the Data Collection Form for Cochrane Reviews [33]. Disagreements during this screening phase will be resolved through discussion with a senior author. For each stage of the review process, agreement will be measured through a weighted kappa statistic.

The eligibility criteria will be pilot tested on a sample of citations at both stages of screening. This will ensure that both reviewers understand the eligibility criteria and allow for refinement if needed. These sets of citations will not be included in the calculation of overall agreement at the end of full-text screening.

### Data collection

A standardized data abstraction document will be developed based on the Data Collection Form for Cochrane Reviews [33]. Two independent reviewers will extract data for each included study. Any discrepancies will be resolved through discussion with the reviewers and a senior author. If necessary, corresponding authors of the eligible studies will be contacted. Where possible, data will be extracted for the intent-to-treat analyses.

All abstracted data will be collected and organized into a MS Excel spreadsheet.

### Data items

For each included study, information related to the study characteristics, patient population, and outcomes of HF care assessed will be extracted. Study characteristics will include study design, component(s) of care assessed in the comparison groups, length of follow-up, setting (i.e., hospital, home, community), sample size, year of study conduct, year of publication, and country of origin. Data regarding the patient population will include age, gender, and severity of HF. In addition, the following outcomes will be extracted where applicable: all-cause and HF-related mortality, all-cause and HF-related hospitalizations, all-cause and HF-related emergency department visits, and health-related quality of life.

### Risk of bias and quality assessment

We will use the modified Cochrane risk of bias tool [34] to assess the risk of bias in each included randomized controlled trial (RCT) in the following domains: random sequence generation, allocation concealment, blinding of outcome assessment, incomplete outcome data, and selective reporting. The Cochrane tool includes a domain to assess blinding of patients or participants; however, for the purpose of this study, this domain is not relevant due to the procedural nature of the intervention(s). Through the Cochrane bias tool, each reviewer assigns a judgment of “high,” “low,” or “unclear” risk for each domain item.

The Risk Of Bias In Non-randomized Studies of Interventions (ROBINS-I) tool will be used to assess bias in each included non-randomized controlled trial or observational study [35] in the following domains: confounding; participant selection; intervention classification; deviations from intended intervention; missing data, including loss to follow-up or missing data on intervention status or other important variables; outcome measurement; and selection of reported results. Risk of bias for each domain is categorized as “low,” “moderate,” “serious,” or “critical.”

Two reviewers will independently assess bias for each included study using the appropriate tool. Any disagreements will be resolved by discussion with a senior author.

### Strength of the evidence assessment

Two reviewers will independently assess the quality of evidence using the Grades of Recommendation, Assessment, Development, and Evaluation (GRADE) system [36] for each included study. We will take into account the risk of bias at the study level, inconsistency, imprecision, indirectness, publication bias, gradient response, large effect, and bias against a significant association [37–42]. Confidence will be rated as high, moderate, low, or very low [43], and disagreements will be resolved by discussion with a senior author. Funnel plots will be used to assess publication bias [44, 45].

### Synthesis of results

The data extracted from the included RCTs will be analyzed separately from the non-randomized controlled trials and cohort studies using network meta-analytic (NMA) techniques to allow us to contrast findings between these study designs, comparing their effectiveness in an ideal context versus in a pragmatic, real-world setting.

In an approach similar to previous work evaluating the effectiveness of different components of psychological care for coronary heart disease [46], four increasingly complex models of the effects of the core components of HF clinic care will be fit for each outcome:

1. Single-effect model. All HF clinic care components will be grouped together as a single treatment, estimating the overall effect of HF clinic care. This is similar to traditional meta-analytic methods that compare HF clinic care to standard care.
2. Additive main effects model. This assumes a separate treatment effect for each of the HF clinic care components. The effect of having two or more components is additive. Each arm of each trial will be coded based on the presence or absences of the particular care component. This decomposes the effect in any given arm into the sum of effects of the components present in that arm.
3. Two-way interaction model. This is an extension of the main effects model that allows for synergistic or antagonistic effects of combinations of each pair of the components of HF clinic care. For example, the effect of component X (e.g., education and counseling) and Y (e.g., clinical monitoring) is not simply the effect of X + Y but could be larger or smaller.
4. Full interaction model. This model assumes that each possible combination of the core components has a distinct effect that cannot be computed from their individual effects or the sums of the two-way interactions. In practice, this model assumes that each different combination of HF clinic care components is a different intervention.

We will choose the simplest model that is consistent with the data and choose between models using the deviance information criterion (DIC). The hazard ratio (HR) and its 95% credible interval (CrI) for each core HF clinic component will be estimated for the mortality, hospitalizations, and emergency department visits outcomes using Markov chain Monte Carlo (MCMC) simulations implemented in JAGS software run through R [47]. For health- related quality of life outcomes, we will estimate the median difference and its 95% CrI. We will run three parallel MCMC chains with a sufficiently large number of iterations and ensure convergence using Gelman-Rubin diagnostic trace plots. Heterogeneity and model fit of the NMA will be assessed through standard approaches [48, 49]. Posterior predictive checks will also be used to identify outlying observations (arms or trials) [50] in the models examining core components. This simulation framework will allow for the presentation of key data summaries of clinical and policy interest, such as the probability that a particular component of care is the most effective for each outcome and the estimated treatment effects for any specified combination of care components.

As we hypothesize that the effectiveness of HF clinic care may differ across a variety of factors, we will explore any potential causes of heterogeneity through the following subgroup analyses, conditional upon data availability: gender, follow-up period, HF severity, method of enrollment and referral (i.e., self-report, systematic referral, or other processes), setting, and number of sites. In addition, as the intensity of care components may vary between studies (i.e., providing one education and counseling session versus providing five sessions), we will define a subjective measure of “intensity” for each component of care, as agreed upon by the study team, and perform stratified analyses to assess the impact of component intensity on effectiveness. We will also consider the country and year of study as standard care may differ between countries and changed over time (i.e., the introduction of new medications and medication protocols in 2016 [51]). Sensitivity analyses including and excluding low-quality studies will be conducted to assess the effect of study quality on our findings.

## Discussion

The global burden of HF is enormous, affecting approximately 26 million people worldwide [52] and reaching yearly global costs of $108 billion [53]. While HF clinic care has been proven beneficial for patients, they have been implemented in widely different formats [8, 20, 21]. Unfortunately, this heterogeneity may considerably undermine their effectiveness [19]. Given limited healthcare resources, identifying the parts of HF clinic care that are associated with better outcomes may allow not only improved care but also reduced costs. As such, it is crucial to understand which components of care offered in HF clinics are the most effective and central to quality patient care.

Studies have attempted to partially address this via traditional systematic review and meta-analysis; for example, a recent Cochrane systematic review and meta-analysis of 25 trials assessed the effectiveness of disease management interventions for patients with HF [30]. The authors note that they were unable to determine the optimal components of HF management programs; however, the study was not designed to address this important question. We believe that the use of network meta-analysis will allow us to identify which components of HF clinic care are associated with improved outcomes.

Care offered to patients in HF clinics is an example of a complex intervention that involves multiple components and multidisciplinary healthcare providers [54–57]. Traditional approaches for evidence synthesis, such as meta-analysis and meta-regression, are not sufficient to handle the assessment of complex health interventions such as HF clinic care [58, 59]. Traditional meta-analysis works well with simple interventions but is unable to separate the effects of individual components of complex interventions. NMA not only allows information from indirect comparisons to be integrated with head-to-head comparisons used in traditional meta-analysis, but will also allow us to model the effects of individual components of HF clinic care. Most commonly based on a framework of Bayesian statistics, NMA has been developed to assess the relative effectiveness of several interventions, synthesizing evidence across a network of randomized trials and observational studies [60–66]. The method simultaneously analyzes direct evidence (studies that directly compare the interventions of interest) and indirect evidence (studies that compare interventions of interest through a common comparator) [67–70], allowing comparison of interventions that have not been evaluated head-to-head. Increasingly, NMA is being used [46, 69, 71–75] to answer questions such as ‘Which type of intervention has the greatest possibility of being most effective?’ or ‘Which combination of components are likely to be most effective?’. As such, we feel that a NMA is a more appropriate method to address our research question.

To our knowledge, this will be the first study to evaluate the comparative effectiveness of the different components of care offered in HF clinics. The findings of this systematic review and network meta-analysis may inform HF guidelines and recommendations regarding the key components of HF clinic care. While referral to and enrollment in HF clinics are currently low, with only one-seventh of HF patients being referred [20], we anticipate growing referral rates as the aging population increases. With limited resources for healthcare, it is critical to understand which components of care yield the best outcomes.

## Additional files

Additional file 1: PRISMA checklist. (PDF 218 kb)

Additional file 2: Preliminary search strategy for Medline. (DOCX 30 kb)

Additional file 3: Study eligibility form. (DOCX 31 kb)

## Abbreviations

CDSR: Cochrane Database of Systematic Reviews
CPCI-S: Conference Proceedings Citation Index-Science
CrI: Credible interval
CRT: Cardiac resynchronization therapy
DARE: Database of Abstracts of Reviews of Effectiveness
DIC: Deviance information criterion
GRADE: Grades of Recommendation, Assessment, Development, and Evaluation
HF: Heart failure
HR: Hazard ratio
HRQoL: Health-related quality of life
ICD: Implantable cardioverter defibrillator
ICTRP: International Clinical Trials Registry Platform
MCMC: Markov chain Monte Carlo
NMA: Network meta-analysis
PRISMA: Preferred Reporting Items for Systematic Reviews and Meta-analyses
PRISMA-P: Preferred Reporting Items for Systematic Reviews and Meta-analysis Protocols
RCT: Randomized controlled trials
SCI-EXPANDED: Science Citation Index Expanded

## References

[1] Ross H, Howlett J, Arnold JM, Liu P, O'Neill BJ, Brophy JM, Simpson CS, Sholdice MM, Knudtson M, Ross DB (2006). Treating the right patient at the right time: access to heart failure care. Can J Cardiol.

[2] Blais C, Dai S, Waters C, Robitaille C, Smith M, Svenson LW, Reimer K, Casey J, Puchtinger R, Johansen H (2014). Assessing the burden of hospitalized and community-care heart failure in Canada. Can J Cardiol.

[3] Zannad F, Agrinier N, Alla F (2009). Heart failure burden and therapy. Europace.

[4] Ho KK, Pinsky JL, Kannel WB, Levy D (1993). The epidemiology of heart failure: the Framingham Study. J Am Coll Cardiol.

[5] Lee WC, Chavez YE, Baker T, Luce BR (2004). Economic burden of heart failure: a summary of recent literature. Heart Lung.

[6] Wijeysundera HC, Machado M, Wang X, Van Der Velde G, Sikich N, Witteman W, Tu JV, Lee DS, Goodman SG, Petrella R (2010). Cost-effectiveness of specialized multidisciplinary heart failure clinics in Ontario, Canada. Value Health.

[7] Beattie WS, Wijeysundera DN (2014). The growing burden of perioperative heart failure. Anesth Analg.

[8] Wijeysundera HC, Trubiani G, Abrahamyan L, Mitsakakis N, Witteman W, Paulden M, van der Velde G, Kingsbury K, Krahn M (2012). Specialized multi-disciplinary heart failure clinics in Ontario, Canada: an environmental scan. BMC Health Serv Res.

[9] Canadian Institute for Health Information (CIHI) (2012). All-cause readmission to acute care and return to the emergency department.

[10] Krumholz HM, Merrill AR, Schone EM, Schreiner GC, Chen J, Bradley EH, Wang Y, Wang Y, Lin Z, Straube BM (2009). Patterns of hospital performance in acute myocardial infarction and heart failure 30-day mortality and readmission. Circ Cardiovasc Qual Outcomes.

[11] Tran DT, Ohinmaa A, Thanh NX, Howlett JG, Ezekowitz JA, McAlister FA, Kaul P (2016). The current and future financial burden of hospital admissions for heart failure in Canada: a cost analysis. CMAJ Open.

[12] Arnold JM, Liu P, Demers C, Dorian P, Giannetti N, Haddad H, Heckman GA, Howlett JG, Ignaszewski A, Johnstone DE (2006). Canadian Cardiovascular Society consensus conference recommendations on heart failure 2006: diagnosis and management. Can J Cardiol.

[13] McAlister FA, Stewart S, Ferrua S, McMurray JJ (2004). Multidisciplinary strategies for the management of heart failure patients at high risk for admission: a systematic review of randomized trials. J Am Coll Cardiol.

[14] Gohler A, Januzzi JL, Worrell SS, Osterziel KJ, Gazelle GS, Dietz R, Siebert U (2006). A systematic meta-analysis of the efficacy and heterogeneity of disease management programs in congestive heart failure. J Card Fail.

[15] Roccaforte R, Demers C, Baldassarre F, Teo KK, Yusuf S (2005). Effectiveness of comprehensive disease management programmes in improving clinical outcomes in heart failure patients. A meta-analysis. Eur J Heart Fail.

[16] Whellan DJ (2005). Heart failure disease management: implementation and outcomes. Cardiol Rev.

[17] Holland R, Battersby J, Harvey I, Lenaghan E, Smith J, Hay L (2005). Systematic review of multidisciplinary interventions in heart failure. Heart.

[18] Thomas R, Huntley A, Mann M, Huws D, Paranjothy S, Elwyn G, Purdy S (2013). Specialist clinics for reducing emergency admissions in patients with heart failure: a systematic review and meta-analysis of randomised controlled trials. Heart.

[19] Jaarsma T, Stromberg A (2014). Heart failure clinics are still useful (more than ever?). Can J Cardiol.

[20] Gravely S, Ginsburg L, Stewart DE, Mak S, Grace SL, Cardiac Rehabilitation Care Continuity Through Automatic Referral Evaluation I (2012). Referral and use of heart failure clinics: what factors are related to use?. Can J Cardiol.

[21] Jaarsma T, Stromberg A, De Geest S, Fridlund B, Heikkila J, Martensson J, Moons P, Scholte op Reimer W, Smith K, Stewart S, Thompson DR (2006). Heart failure management programmes in Europe. Eur J Cardiovasc Nurs.

[22] Jaarsma T, van der Wal MH, Lesman-Leegte I, Luttik ML, Hogenhuis J, Veeger NJ, Sanderman R, Hoes AW, van Gilst WH, Lok DJ (2008). Effect of moderate or intensive disease management program on outcome in patients with heart failure: Coordinating Study Evaluating Outcomes of Advising and Counseling in Heart Failure (COACH). Arch Intern Med.

[23] Angermann CE, Stork S, Gelbrich G, Faller H, Jahns R, Frantz S, Loeffler M, Ertl G, Competence Network Heart F (2012). Mode of action and effects of standardized collaborative disease management on mortality and morbidity in patients with systolic heart failure: the Interdisciplinary Network for Heart Failure (INH) study. Circ Heart Fail.

[24] Stewart S, Carrington MJ, Marwick TH, Davidson PM, Macdonald P, Horowitz JD, Krum H, Newton PJ, Reid C, Chan YK, Scuffham PA (2012). Impact of home versus clinic-based management of chronic heart failure: the WHICH? (Which Heart Failure Intervention Is Most Cost-Effective & Consumer Friendly in Reducing Hospital Care) multicenter, randomized trial. J Am Coll Cardiol.

[25] Chandler J, Hopewell S (2013). Cochrane methods--twenty years experience in developing systematic review methods. Syst Rev.

[26] Moher D, Liberati A, Tetzlaff J, Altman DG, Group P (2009). Preferred reporting items for systematic reviews and meta-analyses: the PRISMA statement. J Clin Epidemiol.

[27] Moher D, Shamseer L, Clarke M, Ghersi D, Liberati A, Petticrew M, Shekelle P, Stewart LA, Group P-P (2015). Preferred reporting items for systematic review and meta-analysis protocols (PRISMA-P) 2015 statement. Syst Rev.

[28] Shamseer L, Moher D, Clarke M, Ghersi D, Liberati A, Petticrew M, Shekelle P, Stewart LA, Group P-P (2015). Preferred reporting items for systematic review and meta-analysis protocols (PRISMA-P) 2015: elaboration and explanation. BMJ.

[29] Hutton B, Salanti G, Caldwell DM, Chaimani A, Schmid CH, Cameron C, Ioannidis JP, Straus S, Thorlund K, Jansen JP (2015). The PRISMA extension statement for reporting of systematic reviews incorporating network meta-analyses of health care interventions: checklist and explanations. Ann Intern Med.

[30] Takeda A, Taylor SJ, Taylor RS, Khan F, Krum H, Underwood M. Clinical service organisation for heart failure. Cochrane Database Syst Rev. 2012;(9):CD002752. 10.1002/14651858.CD002752.pub3 22972058

[31] Howlett JG, McKelvie RS, Costigan J, Ducharme A, Estrella-Holder E, Ezekowitz JA, Giannetti N, Haddad H, Heckman GA, Herd AM (2010). The 2010 Canadian Cardiovascular Society guidelines for the diagnosis and management of heart failure update: heart failure in ethnic minority populations, heart failure and pregnancy, disease management, and quality improvement/assurance programs. Can J Cardiol.

[32] Yancy CW, Jessup M, Bozkurt B, Butler J, Casey DE, Drazner MH, Fonarow GC, Geraci SA, Horwich T, Januzzi JL (2013). 2013 ACCF/AHA guideline for the management of heart failure: executive summary: a report of the American College of Cardiology Foundation/American Heart Association Task Force on practice guidelines. Circulation.

[33] Data collection forms for intervention reviews [https://training.cochrane.org/interactivelearning/module-4-selecting-studies-and-collecting-data].

[34] Higgins JP, Altman DG, Gotzsche PC, Juni P, Moher D, Oxman AD, Savovic J, Schulz KF, Weeks L, Sterne JA (2011). The Cochrane Collaboration's tool for assessing risk of bias in randomised trials. BMJ.

[35] Sterne JA, Hernan MA, Reeves BC, Savovic J, Berkman ND, Viswanathan M, Henry D, Altman DG, Ansari MT, Boutron I (2016). ROBINS-I: a tool for assessing risk of bias in non-randomised studies of interventions. BMJ.

[36] Guyatt GH, Oxman AD, Vist GE, Kunz R, Falck-Ytter Y, Alonso-Coello P, Schunemann HJ, Group GW (2008). GRADE: an emerging consensus on rating quality of evidence and strength of recommendations. BMJ.

[37] Guyatt GH, Oxman AD, Kunz R, Brozek J, Alonso-Coello P, Rind D, Devereaux PJ, Montori VM, Freyschuss B, Vist G (2011). GRADE guidelines 6. Rating the quality of evidence--imprecision. J Clin Epidemiol.

[38] Guyatt GH, Oxman AD, Kunz R, Woodcock J, Brozek J, Helfand M, Alonso-Coello P, Falck-Ytter Y, Jaeschke R, Vist G (2011). GRADE guidelines: 8. Rating the quality of evidence--indirectness. J Clin Epidemiol.

[39] Guyatt GH, Oxman AD, Kunz R, Woodcock J, Brozek J, Helfand M, Alonso-Coello P, Glasziou P, Jaeschke R, Akl EA (2011). GRADE guidelines: 7. Rating the quality of evidence--inconsistency. J Clin Epidemiol.

[40] Guyatt GH, Oxman AD, Montori V, Vist G, Kunz R, Brozek J, Alonso-Coello P, Djulbegovic B, Atkins D, Falck-Ytter Y (2011). GRADE guidelines: 5. Rating the quality of evidence--publication bias. J Clin Epidemiol.

[41] Guyatt GH, Oxman AD, Sultan S, Glasziou P, Akl EA, Alonso-Coello P, Atkins D, Kunz R, Brozek J, Montori V (2011). GRADE guidelines: 9. Rating up the quality of evidence. J Clin Epidemiol.

[42] Guyatt GH, Oxman AD, Vist G, Kunz R, Brozek J, Alonso-Coello P, Montori V, Akl EA, Djulbegovic B, Falck-Ytter Y (2011). GRADE guidelines: 4. Rating the quality of evidence--study limitations (risk of bias). J Clin Epidemiol.

[43] Iorio A, Spencer FA, Falavigna M, Alba C, Lang E, Burnand B, McGinn T, Hayden J, Williams K, Shea B (2015). Use of GRADE for assessment of evidence about prognosis: rating confidence in estimates of event rates in broad categories of patients. BMJ.

[44] Light RJ, Pillemer DB (1984). Summing up: the science of reviewing research.

[45] Egger M, Davey Smith G, Schneider M, Minder C (1997). Bias in meta-analysis detected by a simple, graphical test. BMJ.

[46] Welton NJ, Caldwell DM, Adamopoulos E, Vedhara K (2009). Mixed treatment comparison meta-analysis of complex interventions: psychological interventions in coronary heart disease. Am J Epidemiol.

[47] Plummer M (2016). rjags: Bayesian graphical models using MCMC.

[48] Dias S, Sutton AJ, Welton NJ, Ades AE (2013). Evidence synthesis for decision making 3: heterogeneity--subgroups, meta-regression, bias, and bias-adjustment. Med Decis Mak.

[49] Dias S, Welton NJ, Sutton AJ, Caldwell DM, Lu G, Ades AE (2013). Evidence synthesis for decision making 4: inconsistency in networks of evidence based on randomized controlled trials. Med Decis Mak.

[50] Zhang J, Fu H, Carlin BP (2015). Detecting outlying trials in network meta-analysis. Stat Med.

[51] Yancy CW, Jessup M, Bozkurt B, Butler J, Casey DE, Colvin MM, Drazner MH, Filippatos G, Fonarow GC, Givertz MM (2016). 2016 ACC/AHA/HFSA focused update on new pharmacological therapy for heart failure: an update of the 2013 ACCF/AHA guideline for the management of heart failure: a report of the American College of Cardiology/American Heart Association Task Force on Clinical Practice Guidelines and the Heart Failure Society of America. J Am Coll Cardiol.

[52] Ambrosy AP, Fonarow GC, Butler J, Chioncel O, Greene SJ, Vaduganathan M, Nodari S, Lam CSP, Sato N, Shah AN, Gheorghiade M (2014). The global health and economic burden of hospitalizations for heart failure: lessons learned from hospitalized heart failure registries. J Am Coll Cardiol.

[53] Cook C, Cole G, Asaria P, Jabbour R, Francis DP (2014). The annual global economic burden of heart failure. Int J Cardiol.

[54] Davidson PM, Newton PJ, Tankumpuan T, Paull G, Dennison-Himmelfarb C (2015). Multidisciplinary management of chronic heart failure: principles and future trends. Clin Ther.

[55] Campbell M, Fitzpatrick R, Haines A, Kinmonth AL, Sandercock P, Spiegelhalter D, Tyrer P (2000). Framework for design and evaluation of complex interventions to improve health. BMJ.

[56] Craig P, Dieppe P, Macintyre S, Michie S, Nazareth I, Petticrew M, Medical Research Council G (2008). Developing and evaluating complex interventions: the new Medical Research Council guidance. BMJ.

[57] Craig P, Petticrew M (2013). Developing and evaluating complex interventions: reflections on the 2008 MRC guidance. Int J Nurs Stud.

[58] Greco T, Zangrillo A, Biondi-Zoccai G, Landoni G (2013). Meta-analysis: pitfalls and hints. Heart Lung Vessel.

[59] Mitka M (2010). US government kicks off program for comparative effectiveness research. JAMA.

[60] Lu G, Ades AE (2004). Combination of direct and indirect evidence in mixed treatment comparisons. Stat Med.

[61] Lu G, Ades AE (2006). Assessing evidence inconsistency in mixed treatment comparisons. J Am Stat Assoc.

[62] Salanti G, Higgins JP, Ades AE, Ioannidis JP (2008). Evaluation of networks of randomized trials. Stat Methods Med Res.

[63] Higgins JP, Whitehead A (1996). Borrowing strength from external trials in a meta-analysis. Stat Med.

[64] Ades AE, Sculpher M, Sutton A, Abrams K, Cooper N, Welton N, Lu G (2006). Bayesian methods for evidence synthesis in cost-effectiveness analysis. Pharmacoeconomics.

[65] Caldwell DM, Ades AE, Higgins JP (2005). Simultaneous comparison of multiple treatments: combining direct and indirect evidence. BMJ.

[66] Jansen JP, Fleurence R, Devine B, Itzler R, Barrett A, Hawkins N, Lee K, Boersma C, Annemans L, Cappelleri JC (2011). Interpreting indirect treatment comparisons and network meta-analysis for health-care decision making: report of the ISPOR Task Force on Indirect Treatment Comparisons Good Research Practices: part 1. Value Health.

[67] Mills EJ, Ioannidis JP, Thorlund K, Schunemann HJ, Puhan MA, Guyatt GH (2012). How to use an article reporting a multiple treatment comparison meta-analysis. JAMA.

[68] Mills EJ, Thorlund K, Ioannidis JP (2013). Demystifying trial networks and network meta-analysis. BMJ.

[69] Greco T, Biondi-Zoccai G, Saleh O, Pasin L, Cabrini L, Zangrillo A, Landoni G (2015). The attractiveness of network meta-analysis: a comprehensive systematic and narrative review. Heart Lung Vessel.

[70] Hoaglin DC, Hawkins N, Jansen JP, Scott DA, Itzler R, Cappelleri JC, Boersma C, Thompson D, Larholt KM, Diaz M, Barrett A (2011). Conducting indirect-treatment-comparison and network-meta-analysis studies: report of the ISPOR Task Force on Indirect Treatment Comparisons Good Research Practices: part 2. Value Health.

[71] Roberts KA, Dixon-Woods M, Fitzpatrick R, Abrams KR, Jones DR (2002). Factors affecting uptake of childhood immunisation: a Bayesian synthesis of qualitative and quantitative evidence. Lancet.

[72] Palmerini T, Benedetto U, Bacchi-Reggiani L, Della Riva D, Biondi-Zoccai G, Feres F, Abizaid A, Hong MK, Kim BK, Jang Y (2015). Mortality in patients treated with extended duration dual antiplatelet therapy after drug-eluting stent implantation: a pairwise and Bayesian network meta-analysis of randomised trials. Lancet.

[73] Palmerini T, Biondi-Zoccai G, Della Riva D, Stettler C, Sangiorgi D, D'Ascenzo F, Kimura T, Briguori C, Sabate M, Kim HS (2012). Stent thrombosis with drug-eluting and bare-metal stents: evidence from a comprehensive network meta-analysis. Lancet.

[74] Simpson SH, Lee J, Choi S, Vandermeer B, Abdelmoneim AS, Featherstone TR (2015). Mortality risk among sulfonylureas: a systematic review and network meta-analysis. Lancet Diabetes Endocrinol.

[75] Bucher HC, Guyatt GH, Griffith LE, Walter SD (1997). The results of direct and indirect treatment comparisons in meta-analysis of randomized controlled trials. J Clin Epidemiol.

