# Supplementary material for: Comparative effectiveness of the different components of care provided in heart failure clinics—protocol for a systematic review and network meta-analysis
Source: Syst Rev. 2019 Feb 2;8:40. doi: 10.1186/s13643-019-0953-4 (PMC6359805; doi:10.1186/s13643-019-0953-4)
Supplement: Supplementary file 2 — Preliminary search strategy for Medline. (DOCX 30 kb) [file 13643_2019_953_MOESM2_ESM.docx]

**Preliminary search strategy for Medline**

| **#** | **Searches** |
| --- | --- |
| 1 | heart failure/ or dyspnea, paroxysmal/ or edema, cardiac/ or heart failure, diastolic/ or heart failure, systolic/ or cardio-renal syndrome/ or (((heart or cardiac or myocardial) adj3 (failure or decompensation or edema?)) or (dyspnea adj2 paroxysmal) or (("cardio renal" or cardiorenal or cardio-renal or renocardiac or reno-cardiac) adj3 syndrome?)).ab,ti. |
| 2 | interdisciplinary communication/ or ambulatory care facilities/ or ambulatory care/ or community health centers/ or outpatient clinics, hospital/ or exp patient care team/ or patient education as topic/ or patient medication knowledge/ or medication reconciliation/ or medication adherence/ or medication therapy management/ or exp patient care planning/ or patient care management/ or patient care/ or nursing/ or disease management/ or remote consultation/ or telerehabilitation/ or counselling/ or exp social support/ or self-help groups/ or "referral and consultation"/ or home care services/ or rehabilitation/ or cardiac rehabilitation/ or exp exercise therapy/ or exercise/ or exp physical fitness/ or house calls/ or (((interdisciplin$ or multidisciplin$ or cross-disciplin$ or "cross discipline$" or patient or outpatient or ambulatory or urgent or neighborhood or community or "free standing" or free-standing or satellite) adj5 (medical or health or care) adj5 (facilit$ or team? or center? or clinic?)) or (patient adj3 (education or counselling)) or ((drug or medication) adj3 (optimization or management or reconciliation or adherence or compliance or persistence or nonadherence or noncompliance or non-adherence or non-compliance or "non adherence" or "non compliance")) or (patient adj3 (medication or drug) adj3 knowledge) or (care adj3 (planning or goal? or manag$)) or ((home or clinic) adj3 (assess$ or call? or visit?)) or (home adj5 (care or intervention)) or (disease adj3 management?) or nurs$ or teleconsult$ or ((remote or virtual) adj3 (consult$ or rehabilitation?)) or telerehabilitation? or tele-rehabilitation? or "tele rehabilitation" or homecare or ((psychosocial or social) adj3 (support or network)) or (("self help" or self-help or support) adj3 (group? or club)) or gatekeep$ or referral? or exercise or (physical adj4 (activity or train$ or fit$)) or ((strength or aerobic or resistance) adj4 train$)).ab,ti. |
| 3 | 1 and 2 |
| 4 | randomized controlled trials as topic/ or randomized controlled trial/ or random allocation/ or random allocation/ or double blind method/ or single blind method/ or clinical trial/ or exp clinical trials as topic/ or placebos/ or ((clinic$ adj trial$1) or ((singl$ or doubl$ or treb$ or tripl$) adj (blind$3 or mask$3)) or placebo$ or randomly allocated or (allocated adj2 random)).tw. or clinical trial, phase i.pt. or clinical trial, phase ii.pt. or clinical trial, phase iii.pt. or clinical trial, phase iv.pt. or controlled clinical trial.pt. or randomized controlled trial.pt. or multicenter study.pt. or clinical trial.pt. |
| 5 | 3 and 4 |
| 6 | limit 5 to "all adult (19 plus years)" |
| 7 | animals/ not (animals/ and humans/) |
| 8 | 6 not 7 |
| 9 | letter/ or historical article/ or case report.tw. or review of reported cases.pt. or review, multicase.pt. |
| **10** | **8 not 9** |
| 11 | epidemiologic studies/ or case-control studies/ or control groups/ or matched-pair analysis/ or cross-sectional studies/ or prevalence/ or cohort studies/ or longitudinal studies/ or follow-up studies/ or prospective studies/ or retrospective studies/ or interviews as topic/ or focus groups/ or narration/ or qualitative research/ or ((case* adj5 control*) or (case adj3 comparison*) or (case$ adj5 series) or control group* or cross-sectional or prevalence or transversal or cohort or longitudinal or prospective or retrospective or (("semi-structured" or semistructured or unstructured or informal or "in-depth" or indepth or "face-to-face" or structured or guide) adj3 (interview* or discussion* or questionnaire*)) or focus group* or qualitative or ethnograph* or fieldwork or "field work" or "key informant").tw. |
| 12 | 3 and 11 |
| 13 | limit 12 to "all adult (19 plus years)" |
| 14 | 13 not 7 |
| **15** | **14 not 9** |
