# Supplementary material for: Comparative effectiveness of the different components of care provided in heart failure clinics—protocol for a systematic review and network meta-analysis
Source: Syst Rev. 2019 Feb 2;8:40. doi: 10.1186/s13643-019-0953-4 (PMC6359805; doi:10.1186/s13643-019-0953-4)
Supplement: Supplementary file 3 — Study eligibility form. (DOCX 31 kb) [file 13643_2019_953_MOESM3_ESM.docx]

**Study eligibility form**

| **Article ID:** | | | |
| --- | --- | --- | --- |
| Author: | Year: | Journal: | Title: |

| **Paper:** |  |  |
| --- | --- | --- |
| - Published ≥1990 | **YES** | **NO** |
|  |  |  |
| **Population:** |  |  |
| - Adults (≥ 18 years old) | **YES** | **NO** |
| - Diagnosis of HF | **YES** | **NO** |
| - Not treated by HF clinic prior to study enrollment | **YES** | **NO** |
|  |  |  |
| **Intervention:** |  |  |
| - Evaluate at least one component (or any combination of components) of HF care | **YES** | **NO** |
|  |  |  |
| **Comparison group:** |  |  |
| - Include at least one comparison group (other type of care or standard care) | **YES** | **NO** |
|  |  |  |
| **Outcomes reported:** |  |  |
| - Any of the following after a follow-up of 30 days or more: - All-cause or HF-related mortality - All-cause or HF-related hospitalizations or emergency department visits - Health-related quality of life | **YES** | **NO** |
|  |  |  |
| **Type of article:** |  |  |
| - Cohort study (retrospective or prospective) or - RCT | **YES** | **NO** |
|  |  |  |
| **Duplicated population:** |  |  |
| - If duplicated, does this study provide new information? - If duplicated, is the study more recent? | **YES** | **NO** |
|  |  |  |
| **Study inclusion:** |  |  |
| - All the answers are YES |  | **INCLUDE** |
| - Any answer is NO |  | **EXCLUDE** |
